# Effect of Cold Plasma on the Microbial Safety and Physicochemical Properties of Functional Supplement Powder

**DOI:** 10.1002/fsn3.71268

**Published:** 2025-11-26

**Authors:** Hadis Amiri, Bahare Shabanpour, Parastoo Pourashouri, Samira Tajiknezhad

**Affiliations:** ^1^ Department of Fisheries Gorgan University of Agricultural Sciences and Natural Resources Gorgan Iran; ^2^ Department of Physics, Faculty of Basic Sciences and Technical Engineering Gonbad Kavos University Gonbad Iran

**Keywords:** cold plasma, encapsulation, functional supplement powder, microbial safety

## Abstract

Cold plasma (CP) was used as an innovative nonthermal approach for the decontamination of a functional supplement powder containing fish oil, hydrolyzed fish protein, and shrimp lipid extract in a liposomal structure—a lipid‐rich food matrix with high sensitivity to marine bioactive compound degradation. The supplement powder underwent CP treatment (air atmospheric) for 10–20 min at 8–12 kV, and its safety and physicochemical properties were investigated. Optimal conditions (20 min, 12 kV) resulted in a significant reduction in psychrotrophic bacteria, mesophilic bacteria, and mold and yeast by 2.88, 2.67, and 0.35 log CFU/g, respectively (*p* < 0.05). CP treatment not only enhanced the microbiological safety of the supplements but also preserved their sensory attributes such as taste, aroma, and flavor. However, CP treatment was associated with minor alterations in color parameters, antioxidant capacity, and lipid oxidation, as evidenced by an increase in peroxide levels (0.52–0.58 meqO_2_/kg fat) and thiobarbituric acid values (1.36–1.65 μM) compared to the control samples (*p* < 0.05). These controlled oxidative changes highlight the potential of cold plasma for extending the shelf life of functional supplements containing heat‐sensitive marine bioactive compounds while maintaining their nutritional and therapeutic value, addressing a key challenge in the functional foods industry.

## Graphical abstract

Effect of cold plasma on the microbial safety of functional supplement powder.

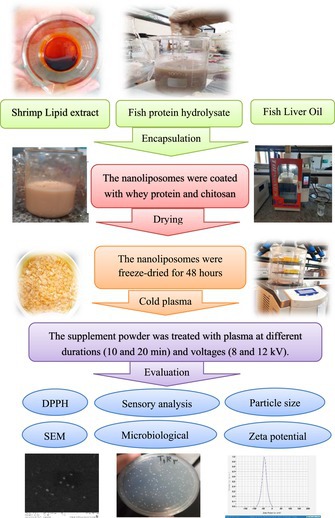

## 1 Introduction

Growing interest in a healthy lifestyle is stimulating consumers to take advantage of functional foods, which can offer higher nutritional benefits. Therefore, such functional foods must possess the sensory and food safety characteristics required by the market for competition and consumer acceptance (Balivo et al. 2024; Gómez‐Mascaraque et al. 2022). Functional supplements containing marine bioactive compounds derived from fish and crustacean by‐products, such as fish oil, fish protein hydrolysate, and shrimp lipid extract, are susceptible to contamination by microorganisms like coliforms, molds, and yeasts during production, processing, or storage. These microorganisms can affect the quality, safety, and sensory properties of the final product. Given the potential risks of foodborne illnesses, the global food industry is prioritizing the development of more effective decontamination techniques to ensure product safety (Jayasena et al. 2015).

Recently, novel nonthermal preservation methods that provide optimum conditions for specific needs against microbial growth have been developed. CP, a cutting‐edge nonthermal technology, has the potential to inactivate a wide range of microorganisms, including fungi, spores, viruses, and bacteria (Olatunde et al. 2020), making it superior to other thermal preservation methods for temperature‐sensitive marine bioactive compounds. By ionizing gas at room temperature, CP generates reactive oxygen and nitrogen species, UV radiation, and charged particles that efficiently deactivate microorganisms through oxidative damage to lipids, proteins, and nucleic acids (Calvo et al. 2016; Liao et al. 2017; Tappi et al. 2016; Darvish et al. 2022).

Previous studies have demonstrated the potential of CP for microbial inactivation in plant‐based powders and liquid foods, including spirulina powder (Beyrer et al. 2020), turmeric (Hemmati et al. 2021), green tea powder (Hemmati et al. 2021), saffron stigma (Birjandi Toroghi et al. 2024), and apple juice (Jiao et al. 2018). These investigations also showed that the impact of CP on bioactive compounds varies with the food matrix and treatment conditions, in some cases reducing antioxidant activity while in others enhancing phenolic content. Additionally, a study conducted by Birjandi Toroghi et al. (2024) revealed that sensory alterations in plasma‐processed foods can differ based on their nutritional attributes such as fatty acid composition, protein content, and fat content. This highlights the matrix‐dependent nature of CP effects, which is particularly relevant when considering marine‐derived functional powders rich in polyunsaturated fatty acids (PUFAs) and specific bioactive peptides.

Despite extensive research on CP in fruits (Chutia and Mahanta 2021), vegetables, and plant‐based powders, no study to date has investigated its application to marine‐derived functional powders. This represents a significant knowledge gap, since these supplements contain highly unsaturated fatty acids and specific marine peptides that may be more susceptible to plasma‐induced oxidation (Amiri et al. 2024).

Therefore, this study aims to evaluate the effects of atmospheric cold plasma treatment at various times and voltages on the physicochemical properties, oxidative stability, and microbial safety of a marine‐derived functional supplement powder. By addressing both microbial inactivation and preservation of nutritional and sensory quality, this work provides the first report on CP application in marine‐based functional powders and offers valuable insights for the functional food industry.

## 2 Materials and Methods

Fish liver oil (*Gadusmorrhua*, Sigma, Germany), crucian carp (*Carassius auratus*), Alcalase enzyme (Novazyme, Denmark), whey protein concentrate (Protein 80%, Welac, UK), soybean lecithin (Sigma, Germany), chitosan (deacetylation degree 85%, medium molecular weight), double‐distilled water, and DPPH (1,1‐diphenyl‐2‐picrylhydrazyl). The other chemical materials were sourced from Merck in Darmstadt, Germany.

### 2.1 Fish Protein Hydrolysate

Crucian carp ( *C. auratus* ) weighing approximately 300 g were obtained from a fish market in Golestan Province, Iran. The fish were beheaded, eviscerated, and the fillets were minced. The mince was defatted with isopropanol in a 1:2 w/v ratio and washed twice with distilled water in a 1:5 w/v proportion. The defatted minced fish was heated with distilled water in a 1:5 w/v ratio in a water bath at 85°C for 20 min and immediately cooled in a cold water/ice mixture to deactivate the endogenous enzymes. Alcalase enzyme (2% of the total fish protein) was added to the samples after adjusting the pH to 8.5 using 0.2 M NaOH, which is the optimal pH for Alcalase activity. Enzymatic hydrolysis was carried out in a shaking incubator at 200 rpm for 2 h at 60°C. The reaction was stopped by heating at 95°C for 20 min in a water bath and then rapidly cooled in an ice‐water mixture. The samples were centrifuged for 20 min at 8000 × *g*. The supernatant was dried for 48 h using a freeze dryer and stored at −20°C until the production of the supplement powder (Amiri et al. 2023; Ovissipour et al. 2013).

### 2.2 Preparation of Shrimp Lipid Extract

The waste of Western White Leg Shrimp ( *Litopenaeus vannamei* ), including the shell, head, and tail, was obtained in a frozen and hygienic state from a shrimp processing factory in Golestan Province, Iran. After thawing, the waste was ground. The homogenized waste (500 g) was mixed with 2500 mL of ethyl acetate and left in a dark environment for 60 min. The sample was filtered using filter paper number 1, and the solvent extraction (ethyl acetate) was carried out using a rotary evaporator. The resulting lipid extract containing astaxanthin pigments was stored in sterile sealed containers at −80°C (Amiri et al. 2023; Gómez‐Mascaraque et al. 2017; Montero et al. 2016).

### 2.3 Preparation of Functional Supplement Powder

The functional supplement powder was created by encapsulating shrimp lipid extract, fish protein hydrolysate, and fish oil in nanoliposomes. These nanoliposomes were coated with whey protein concentrate and chitosan in a bilayer structure. The process involved freezing the nanoliposomes at −80°C and then drying them under vacuum for 48 h using a Labconco FreeZone freeze dryer at −70°C and a pressure of 0.001 mbar. To elaborate, a solution containing fish protein hydrolysate (1% w/w), shrimp lipid extract (0.1% w/w), fish oil (1% w/w), and soybean lecithin (5% w/w) was mixed at 35°C on a magnetic stirrer. After stabilization, the mixture underwent sonication for 8 min at 400 watts and 20°C. The resulting nanoliposomes were stored in the refrigerator before the coating process. The nanoliposomes were coated layer‐by‐layer by combining them with chitosan (0.5%, pH 4.5) and whey protein (10% aqueous solution) through electrostatic deposition. The negatively charged nanoliposomes were mixed with the positively charged chitosan solution for 150 min, followed by the addition of the whey protein solution in equal parts and stirring for 120 min. The bilayer nanoliposomes were then frozen at −80°C and dried under vacuum for 48 h using a freeze dryer (Amiri et al. 2024).

### 2.4 Treatment of Samples With CP

CP was generated using a dielectric barrier discharge (DBD) system consisting of two parallel circular stainless‐steel electrodes (15 cm in diameter). The lower electrode was covered with a 2‐mm thick quartz dielectric plate, and the distance between the two electrodes was fixed at 5 mm using a piston‐controlled mechanism. The system was powered by a sinusoidal AC power supply (50 Hz) with a maximum output voltage of 15 kV and an output power of 150 W; in this study, treatments were carried out at 8 and 12 kV. Air at atmospheric pressure was used as the working gas. Prior to treatment, the plasma generator and handling tools were disinfected with ethanol, and all procedures were performed under a laminar flow hood to minimize contamination. Approximately 5 g of the functional supplement powder was evenly spread on the dielectric‐covered ground electrode to form a thin layer directly exposed to the discharge. Samples were treated at two exposure times (10 and 20 min) under voltage conditions of 8 and 12 kV. Immediately after treatment, powders were transferred to sterile airtight plastic containers and stored at 4°C until further analyses. Plasma‐treated samples were subsequently evaluated for physicochemical characteristics, oxidative stability, microbial safety, and sensory properties.

### 2.5 Morphology, Mean Particle Size, and Zeta Potential

To examine the morphology and surface characteristics of the nanoliposomes using Scanning Electron Microscopy (SEM) (TESCAN, VEGA3), serial dilutions of the nanoliposomes were prepared. A droplet from each dilution was placed and dried onto small glass slide fragments for SEM imaging. Samples were placed on a stub and fixed with a double‐layer sticky coating equipped with a conductive carbon plate. Subsequently, a thin layer of gold was applied for 8 s to enhance the secondary electron signal. The metallized nanoliposomes were then imaged using a focused Ga+ electron beam at an acceleration voltage of 3 kV (current < 6e‐4 Pa) and observed at magnifications of 20,000× and 45,000× (Jiménez‐Martín et al. 2015).

The particle size of the nanoliposomes was determined using dynamic light scattering, and surface charge (zeta potential) was determined through electrophoresis on a DLS instrument (HORIBA, SZ‐100, Japan). All samples were diluted in deionized water (1:100) and analyzed at 25°C. Each sample was analyzed three times for accuracy.

### 2.6 Free Radical Scavenging (DPPH)

To assess the DPPH scavenging activity, the supplement powder (1 g) was diluted with 90% ethanol in a 1:5 ratio and then centrifuged at 10,000 × *g* for 20 min at 4°C. Next, 1 mL of the supernatant was mixed with 2 mL of a DPPH solution (0.2 mM/L in ethanol) and the absorbance was read at 525 nm using a microplate reader (Biochrom, Libras12, UK) after 45 min of incubation in the dark. Distilled water served as the blank, the sample with ethanol as the control, and BHT at 0.2 mg/mL as the positive control. The DPPH scavenging activity was calculated using the formula provided by (Jiao et al. 2018).

(1) $$\text{DPPH}\;\left(\%\right)=1-\left(\left(A-B\right)/\left(C\right)\right)\times 100$$

where *A* = sample absorption; *B* = sample control absorption; *C* = blank absorption.

### 2.7 Color Measurement

The functional supplement powder (5 g) was evenly spread in a Petri dish. Color indices, including brightness (L*), redness (a*), and yellowness (b*), were measured for three randomly selected samples from each treatment using a colorimeter device (Lovibond, CAM‐system, UK) in a controlled environment. A standardized viewing booth with D65 illuminant (daylight, 6500 K) was used to eliminate ambient lighting variations. The instrument was calibrated with standard black and white tiles before analysis (Liu et al. 2016).

### 2.8 Peroxide Value (PV)

To prepare the samples, the test tubes containing treatments (300 μL) were stirred vigorously with 1.5 mL of a 2‐propanol/isooctane mixture (1:3 v/v) for 30 s. The mixture was then centrifuged at 2500 × *g* for 5 min. Next, 200 μL of the organic layer was combined with 2.8 mL of a 1‐butanol/methanol mixture (1:2 v/v). Following this, 30 μL of an ammonium thiocyanate/Fe^+2^ solution (by combining 1 mL of ammonium thiocyanate 3.94 M and 1 mL of freshly prepared Fe^+2^ solution) was added, and the treatment was incubated in the dark for 20 min. The absorbance of the sample was measured at 510 nm after homogenization for 5 s. The blank consisted of all the chemicals except the sample. All analyses were done in triplicate. A standard curve was generated using various concentrations of cumene hydroperoxide, and the hydroperoxide concentration was calculated (0–0.4 meqO_2_/kg fat) (Guner and Oztop 2017).

### 2.9 Secondary Oxidation Products

To analyze the thiobarbituric acid reactive substances (TBARS), 1 mL of the sample was mixed with 2 mL of a TBARS stock solution containing trichloroacetic acid (15% w/v), thiobarbituric acid (0.375% w/v), and hydrochloric acid (0.25 M) in double‐distilled water. The mixture was then heated in a water bath at 95°C for 15 min. After cooling, the samples were centrifuged at 15,000 × *g* for 5 min. The absorbance of the supernatant was measured at 532 nm. Material concentrations were determined using a standard curve (*r* ^2^ = 0.990) created with various concentrations of 1,1,3,3‐tetramethoxypropane (0–20 μL). All analyses were done in triplicate (Gumus et al. 2017).

### 2.10 Sensory Assessment

To conduct sensory evaluation, a functional supplement powder was mixed with milk in a ratio of 1.5–100 g. The mixture was refrigerated until the sensory analysis was performed. A group of 25 consumers aged 20–25, who gave informed consent, took part in the sensory analysis. Following extensive training, each panelist received around 15 g of the milk‐supplement mixture in odorless plastic containers labeled with random three‐digit codes. Water and unsalted crackers were provided for palate cleansing between samples. The panel sessions were held around 2 h before lunchtime. Ten minutes after removing the treatments from the refrigerator, the evaluation was conducted in a well‐lit room with a consistent temperature of 20°C using a hedonic scale. The scale included ratings from 1 to 9 as follows: 1 (extremely disliked or no intensity), 2 (very disliked), 3 (disliked), 4 (slightly disliked), 5 (neither liked nor disliked), 6 (slightly liked), 7 (liked), 8 (very liked), 9 (extremely liked or high intensity). The study was approved by IRB no. 98‐412‐56 (Amiri et al. 2024; Aspevik et al. 2021; Jiménez‐Martín et al. 2016).

### 2.11 Microbiological Analysis

The microbial analysis of the samples involved homogenizing 1 g of the supplement powder under plasma treatment in 9 mL of physiological saline (0.9% NaCl solution) under sterile conditions. After shaking the samples for 30 s, serial dilutions were prepared by transferring 1 mL of the homogenized mixture to a test tube containing 9 mL of sterile physiological saline and homogenizing to achieve a dilution of 0.1. Subsequent dilutions (R1, R2, and R3) were prepared in the same manner. Bacterial cultures were then performed by pouring 0.1 mL of the diluted samples onto sterile disposable plates containing Plate Count Agar (PCA), which consists of casein, peptone, dextrose, and yeast extract agar. Psychrophilic bacteria colonies were counted after 14 days of storage at 4°C, while mesophilic bacteria were counted after 24 h of incubation at 37°C. Mold and yeast colonies were cultured using 0.1 mL of the diluted sample on sterile disposable plates containing YGC medium (Yeast extract Glucose Chloramphenicol Agar). Evaluation of mold and yeast colonies was conducted after 5 days of incubation at 25°C, and for treatments stored in the refrigerator for 1 month. Three replicates of each treatment were cultured, and the microbial load of the treatments was calculated in terms of log CFU (colony‐forming units)/g (Institute of Standards and Industrial Research of Iran 2008).

### 2.12 Statistical Analysis

The data were analyzed using SPSS software, version 16. Normality checks were conducted in a completely randomized design. One‐way analysis of variance (ANOVA) was performed to analyze the results. A significant difference comparison was conducted using ANOVA and Duncan's test. The analytical results are presented as mean ± standard deviation, with a significance level set at *p* < 0.05.

## 3 Results and Discussion

### 3.1 Morphology, Mean Particle Size, and Zeta Potential

**FIGURE 1:**
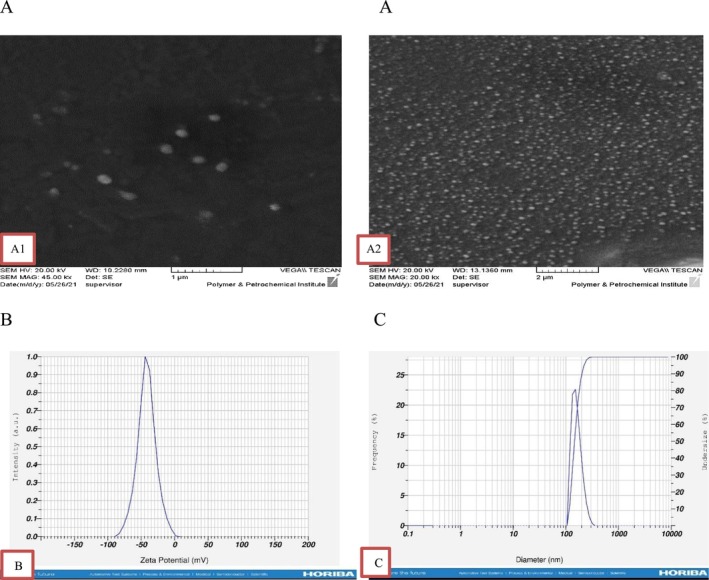
(A) SEM images at a magnification of 25,000**×** and 45,000**×**, (B) mean particle size, and (C) zeta potential of bilayer nanoliposomes.

The morphological analysis of nanoliposomes provides valuable insights into their surface structure and the factors influencing their physical properties. Scanning electron microscopy (SEM) images revealed that the bilayer nanoliposomes, containing shrimp lipid extract, fish protein hydrolysate, and fish oil, had spherical shapes with smooth surfaces, confirming that the produced vesicles are nanoliposomes and not random clusters of phospholipids. The encapsulation efficiency of the nanoliposomes was high at 97.89% ± 0.89%, with no leakage of core material observed (Figure 1A). The mean particle size of bilayer nanoliposomes was 153.7 nm, which is consistent with dynamic light scattering (DLS) data in Figure 1B. The zeta potential of the bilayer nanoliposomes was reported to be −41.8 mV (Figure 1C).

### 3.2 DPPH

**FIGURE 2:**
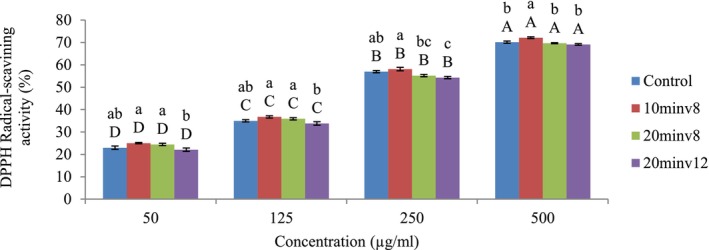
DPPH inhibitory power of free radicals in the functional supplement powder under plasma treatment at different conditions: T1 control sample (no radiation), T2 (10 min at 8 kV), T3 (20 min at 8 kV), and T4 (20 min at 12 kV). Letters a–c indicate a significant difference among various treatments under plasma (*p* < 0.05). Capital letters A–D show a significant difference among treatments at various concentrations (*p* < 0.05).

The inhibitory power of free radicals in a functional supplement powder under plasma treatment and the control sample at various concentrations (500, 250, 125, and 50 μg/mL) is shown in Figure 2. According to the results, the inhibitory effect of free radicals increased with the increasing concentration of treatments (*p* < 0.05). Additionally, increasing the time and voltage of plasma treatment had a significant effect on the inhibitory power of free radicals in the samples (*p* < 0.05). Specifically, plasma treatment (T4) with a time of 20 min and a voltage of 12 kV had less inhibitory power compared to other treatments, but did not show much difference compared to the control sample. Similar results were observed in the study by Birjandi Toroghi et al. (2024) regarding the effect of plasma at different times and power levels on the antioxidant activity of saffron stigmas. Increasing the plasma treatment time to 10 min in an air atmosphere with fixed power up to 100 watts resulted in a decrease in antioxidant activity. This was attributed to the oxidative processes induced by the active radicals generated during plasma treatment. Further research by Hemmati et al. (2021) on green tea powder and Liao et al. (2018) on apple juice showed that increased durations and voltage levels of plasma treatment led to a decrease in antioxidant activity (*p* < 0.05). The findings of this study contradict previous research by Darvish et al. (2022) on onion skin and Jamali et al. (2025) on chia seeds, which reported an increase in antioxidant activity following plasma treatment. They stated that this increase could be attributed to the ultraviolet radiation generated during plasma treatment, which might trigger the production of secondary metabolites. Their study indicated that the impact of plasma treatment on phenolic content and antioxidant activity is dependent on the duration and voltage of the treatment. In a study by Kim et al. (2017), onion powder treated with microwave cold plasma exhibited higher free radical inhibitory activity compared to untreated powder. They mentioned that the antioxidant activity of samples was not reduced by active species present in plasma. Additionally, CP has the potential to prevent contamination of onion powder products without adversely affecting their physicochemical properties and taste. Sanaee et al. (2020) demonstrated that the type of gas had no significant effect on the inhibitory index of turmeric samples. However, the duration of plasma application had a significant impact, leading to a decrease in their antioxidant properties compared to the control sample (*p* < 0.05). Discrepancy in results indicates that the type of food product, plasma production source, and the state of plasma exposure (direct and indirect) to the target substance are vital in controlling the effects of CP on the antioxidant activity of food products (Pankaj et al. 2018).

### 3.3 Peroxide (PV)

Fish oil, hydrolyzed fish protein, and shrimp lipid extract contain various unsaturated fatty acids. Due to the high sensitivity of these unsaturated fatty acids, the risk of oxidation increases. Changes in the peroxide level of the functional supplement powder under plasma treatment at different times and voltages are presented in Table 1. The results show that the peroxide levels of the functional supplement powder were significantly affected by the duration and voltage of plasma treatment (*p* < 0.05). Specifically, samples treated with plasma for 20 min at 12 kV had a higher peroxide level (0.58 meqO_2_/kg fat) compared to other samples. However, all peroxide levels remained below the critical thresholds set for fresh fish oil and functional supplements according to international food standards (PV ≤ 5 meqO_2_/kg fat) (Codex Alimentarius 1991). This significant increase in peroxide values may be attributed to the reactive oxygen species generated by the plasma interacting with the unsaturated fatty acids in fish oil and shrimp lipid components. However, despite this slight increase, the overall acceptance of the functional supplement powder was not significantly affected (*p* < 0.05). These findings contrast with a study by Choi et al. (2016) that reported no significant changes in peroxide values after corona discharge plasma treatment on dried Alaska pollock, possibly due to differences in treatment methods, substrate composition, and exposure duration. In another study by Zouelm et al. (2019), the impact of plasma treatment on the quality and spoilage of Pacific white shrimp ( *L. vannamei* ) during refrigerated storage showed that shrimp treated with CP and metabisulphite had a slower increase in peroxide value and thiobarbituric acid reactive substances compared to the control sample.

**TABLE 1:** Changes in peroxide value (PV), thiobarbituric acid (TBA) value, brightness (L*), redness (a*), and yellowness (b*) of the supplement powder under plasma treatment at various times and voltages.

Treatment	Peroxide (meqO_2_/kg fat)	TBA (μM)	(L*)	(a*)	(b*)
(T1) Control sample	0.52 ± 0.011^b^	1.36 ± 0.04^c^	75.03 ± 0.47^c^	12.27 ± 0.23^a^	12.27 ± 0.23^c^
(T2) 10 min at voltage 8 kV	0.53 ± 0.008^b^	1.34 ± 0.02^c^	75.23 ± 0.24^bc^	11.8 ± 0.17^ab^	12.73 ± 0.09^bc^
(T3) 20 min at voltage 8 kV	0.56 ± 0.005^a^	1.51 ± 0.03^b^	76.1 ± 0.23^ab^	11.53 ± 0.18^b^	13.1 ± 0.32^bc^
(T4) 20 min at voltage 12 kV	0.58 ± 0.005^a^	1.65 ± 0.05^a^	76.73 ± 0.23^a^	11.27 ± 0.19^b^	13.43 ± 0.29^a^

*Note:* Lowercase letters a–d indicate a significant difference among different treatments under plasma radiation (*p* < 0.05).

### 3.4 Thiobarbituric Acid Reactive Substances (TBARS)

The measurement of secondary oxidation products is crucial for assessing the oxidation of fats in food, as they contribute to active odors, unlike primary oxidation products which are colorless and tasteless (Almeida et al. 2015). Table 1 displays the levels of reactive compounds with thiobarbituric acid in functional supplement powder treated with plasma at varying times and voltages. The results indicate that increasing the time and voltage of plasma treatment significantly impacted the levels of reactive compounds with thiobarbituric acid in the functional supplement powder (*p* < 0.05). Specifically, the sample treated with plasma for 20 min at 12 kV exhibited higher levels of secondary oxidation compounds compared to other samples. In contrast, the control sample (without plasma treatment) had lower levels of secondary oxidation compounds. The increase in TBA levels did not have a significant impact on the overall acceptability of the functional supplement powder. All treatments had TBA levels below the critical threshold of 8 mg MDA/kg established for rancidity and off‐flavors in fish products by Secci and Parisi (2016). In a study by Matra et al. (2025), similar findings were reported when CP was applied to salmon patties. These researchers attributed the increase in TBA levels during cold plasma treatment to the oxidation of long‐chain fatty acids in fish oil. They stated that reactive oxygen species, charged particles, and by‐products of protein degradation produced during the plasma treatment could play a role in enhancing the oxidation of the samples. Other studies on olive oil (Van Durme and Vandamme 2016) and pork (Jayasena et al. 2015) also reported an increase in secondary oxidation products following plasma treatment. In a study by Kim et al. (2011), the effect of atmospheric pressure plasma on pathogen inactivation in bacon was investigated using a combination of two different gases. The TBA levels in plasma‐treated bacon varied, with samples treated for 60 and 90 s showing higher thiobarbituric acid values compared to the control sample.

### 3.5 Color Measurement

Table 1 displays the average color parameters of the functional supplement powder under plasma treatment compared to the control sample (without plasma radiation). The brightness (L*) and yellowness (b*) indices of the supplement powder increased significantly with increasing time and voltage of plasma treatment in different cycles (*p* < 0.05). The increase in L* indicates that the samples lightened after plasma treatment. Previous studies have also reported similar effects of plasma treatment on color parameters. For example, Almeida et al. (2015) and Tavakoli Lahijani et al. (2022) explained that CP generates numerous active molecules, such as nitric oxide, peroxide, hydroxyl radicals, and ozone with minimal energy at room temperature. These active molecules can react with the color compounds present in the supplement powder and, due to oxidation, lead to the lightening of the product's color.

In contrast, the redness index (a*) of the supplement powder decreased significantly (*p* < 0.05). The redness index was highest in the control sample and lowest in the plasma treatment with 20 min and 12 kV voltage, suggesting a reduction in astaxanthin pigment content (double conjugated bonds) due to plasma treatment. Similar results about the effect of plasma on the color of paprika and red pepper powder were reported by Hertwig et al. (2015) that the redness index decreased over time, while the brightness and yellowness indices increased. Lee et al. (2016) found that plasma treatment on brown rice samples increased the brightness index but slightly decreased the redness and yellowness indices. Sanaee et al. (2020) investigated the effect of CP on turmeric and observed a decrease in all three color indices with increasing plasma application time, with significant differences at 15 and 25 min (*p* < 0.05).

### 3.6 Sensory Assessment

**FIGURE 3:**
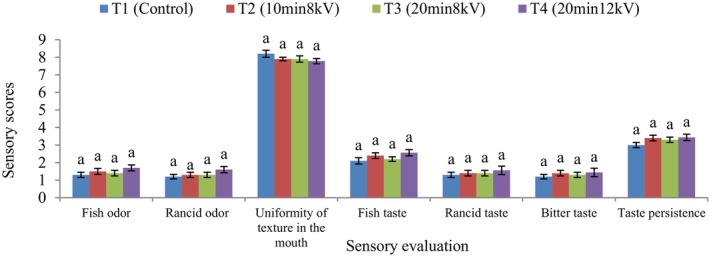
Sensory evaluation indices in milk containing functional supplement powder under plasma treatment at various conditions: T1 control sample (no radiation), T2 (10 min at 8 kV), T3 (20 min at 8 kV), and T4 (20 min at 12 kV). Letters (a) indicate no significant difference among treatments (*p* < 0.05).

The sensory analysis of the functional supplement powder subjected to plasma treatment and control samples (without plasma irradiation) is shown in Figure 3. The results indicate that there were no significant differences in sensory evaluation scores of the milk‐supplement mixture before consumption (fish odor, oxidation odor) and after consumption (uniformity of texture in the mouth, fish taste, bitter taste, oxidation taste, and taste persistence) between the plasma‐treated samples and the control samples (*p* < 0.05). Therefore, increasing the plasma treatment time and voltage at optimal conditions (20 min and 10 kV) preserved the sensory characteristics of the milk‐supplement powder mixture compared to the control sample. Previous studies have also reported similar effects of plasma treatment on sensory characteristics. Basaran et al. (2008) utilized cold plasma (hexafluoride gas and air) for 20 min to deactivate *Aspergillus parasiticus* in nut kernels. They found that the nut kernels treated with plasma did not exhibit any significant difference in terms of color, odor, taste, and overall acceptance compared to control samples. Matan et al. (2015) investigated the impact of atmospheric pressure plasma discharge on the quality indicators of pitaya fruit. The sensory evaluation results indicated no significant difference between the sensory indicators of plasma‐treated samples and the control samples (*p* < 0.05). These results contrast with the findings of Birjandi Toroghi et al. (2024), where prolonged plasma treatment led to a slight reduction in the aroma, flavor, and color of saffron stigmas. Therefore, sensory changes in plasma‐treated foods can vary based on food characteristics like fatty acid composition, protein content, and fat content (Yong et al. 2015).

### 3.7 Microbiological Analysis

#### 3.7.1 Total Bacterial Count

**FIGURE 4:**
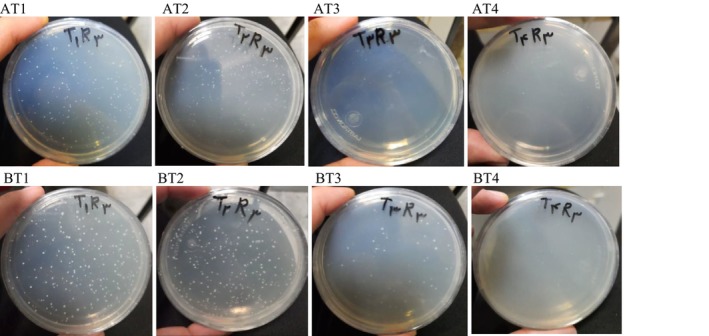
The total bacteria count in the functional supplement powder under plasma treatment at different times and voltages: T1 control sample (no radiation), T2 (10 min at 8 kV), T3 (20 min at 8 kV), and T4 (20 min at 12 kV). (A) Psychrotrophic bacteria after 14 days of storage at 4°C, and (B) mesophilic bacteria after 24 h of incubation at 37°C.

Table 2 displays the microbial load (psychrotrophic bacteria) of the plasma‐treated functional supplement powder compared to the control sample after 14 days of storage at 4°C. The data indicate that increasing the time and voltage of the plasma treatment in various operational cycles resulted in a significant reduction of the total number of microorganisms (*p* < 0.05) (Figure 4A). Notably, the plasma treatment T4 (20 min at 12 kV) reduced the bacterial count by 2.88 log CFU/g, with no microorganisms detected in the sample. Furthermore, Table 2 presents the total count of mesophilic bacteria in the plasma‐treated samples compared to the control sample after 24 h of incubation at 37°C. The results demonstrate a significant decreasing trend in the total mesophilic bacterial count with increasing time and voltage of the plasma treatment (*p* < 0.05) (Figure 4B). Specifically, the plasma treatment T4 (20 min at 12 kV) resulted in a significant decrease in the mesophilic bacterial count from 2.92 to 2.92 log CFU/g. The study's results align with antimicrobial mechanisms described in plasma sterilization literature. The effectiveness of the T4 treatment (20 min at 12 kV) parameters mirrors findings by Roth et al. (2000), who achieved a significant reduction in *Escherichia coli* and *Staphylococcus aureus* populations using a 20‐min CP discharge. Villeger et al. (2003) also reported 100% *E. coli* elimination after a 20‐min discharge with an N_2_–O_2_ gas mixture. Similar results on onion skins under plasma treatment were reported by Senguler et al. (2024), where the total mesophilic aerobic bacteria and yeast‐mold counts decreased after 20 min at 40 kV. In a study by Darvish et al. (2022), the greatest reduction in microbial load of saffron was observed under plasma treatment for 30 min at 110 W. Also, Ziuzina et al. (2014) found that varying durations of plasma treatment led to undetectable levels of microbes on tomatoes. However, our achievement of complete sterilization in a powder matrix represents a notable advancement, as powder systems typically present greater challenges for plasma penetration compared to liquid or surface applications. The proposed antimicrobial mechanisms involving free radicals, ultraviolet radiation, and charged particles likely contributed to our results (Moisan et al. 2001). These charged particles can deactivate bacterial cells by disrupting the cytoplasmic membrane, leading to its rupture. The presence of reactive oxygen and hydroxyl radicals can also damage the cell wall by oxidizing fatty acids and amino acids. These radicals can generate hydrogen peroxide and hydroperoxy radicals, which act as antimicrobial agents with a longer life (Darvish et al. 2022; Laroussi et al. 2003; Surowsky et al. 2014). The successful application of these mechanisms to supplement powder sterilization extends previous work by Nateghi et al. (2024) on potato slices and Deng et al. (2007) on almonds, demonstrating that cold plasma can effectively sterilize diverse food matrices while maintaining the functional properties essential for supplement powder applications.

**TABLE 2:** Total counts of psychrotrophic bacteria (14 days at 4°C), mesophilic bacteria (24 h at 37°C), and mold and yeast (5 days at 25°C) in the supplement powder under plasma treatment at different times and voltages compared to the control sample.

Treatment	Psychrotrophic bacteria (log CFU/g)	Mesophilic bacteria (log CFU/g)	Mold and yeast (log CFU/g)
(T1) Control sample	2.88 ± 0.01^a^	2.92 ± 0.01^a^	0.35 ± 0.05^a^
(T2) Plasma radiation 10 min at 8 kV	2.67 ± 0.02^b^	2.89 ± 0.02^a^	ND^b^
(T3) Plasma radiation 20 min at 8 kV	1.44 ± 0.03^c^	2.36 ± 0.03^b^	ND^b^
(T4) Plasma radiation 20 min at 12 kV	ND^d^	0.25 ± 0.14^c^	ND^b^

*Note:* Values are presented as mean ± standard deviation. Values with different superscript letters in the same column are significantly different (*p* < 0.05).

Abbreviation: ND, not detected.

#### 3.7.2 Mold and Yeast Count

**FIGURE 5:**
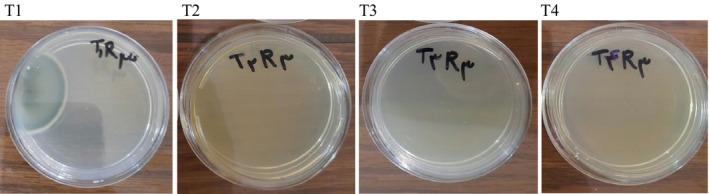
Mold and yeast levels of the functional supplement powder under plasma treatment at different times and voltages, during 5 days of storage at the incubation temperature of 25°C. T1 represents the control sample (no radiation), T2 (10 min at 8 kV), T3 (20 min at 8 kV), and T4 (20 min at 12 kV).

Table 2 presents the mold and yeast levels in the functional supplement powder before and after plasma treatment following 5 days of storage at an incubation temperature of 25°C (Figure 5). The results indicate a significant reduction of 0.35 log CFU/g in mold and yeast levels in the plasma‐treated functional supplement powder at different times and voltages (T2, T3, and T4) compared to the control sample (*p* < 0.05). Notably, no mold or yeast was detected in any of the plasma‐treated samples. Previous studies have demonstrated that CP is effective in inactivating various microorganisms, including molds and yeasts. The findings from Trebulová et al. (2023) confirm the significant inhibitory effects of CP on the yeast *C. glabrata* . They stated that the overall inhibitory effects are directly related to treatment time, applied power, and the overall performance of the device. Darvish et al. (2022) attributed the significant reduction in mold and yeast present in saffron after prolonged plasma treatment to active species, ultraviolet photons, and free radicals, which induce morphological changes through interaction and penetration into the cells of microorganisms. Further research by Basaran et al. (2008) on nut surfaces, Suhem et al. (2013) on agar culture media and brown rice, and Lacombe et al. (2015) on blueberries showed the efficacy of CP treatment in successfully eliminating *A. parasiticus* and inhibiting *Aspergillus flavus*, respectively.

Furthermore, mold and yeast levels in the functional supplement powders under plasma irradiation were also examined for 1 month at refrigeration temperature (4°C). Results showed no mold or yeast was observed in either the plasma‐treated or control samples stored at 4°C.

## 4 Conclusion

This study demonstrated that the CP treatment had a positive effect on the disinfection of a functional supplement powder containing fish oil, hydrolyzed fish protein, and shrimp lipid extract within a lipid‐rich liposomal structure. The optimal CP treatment conditions (20 min at 12 kV) significantly reduced psychrotrophic bacteria, mesophilic bacteria, molds, and yeasts in a time‐ and voltage‐dependent manner, enhancing the microbial safety of the supplement without compromising its sensory and nutritional properties. However, high levels of CP treatment resulted in significant changes in lipid oxidation. The extent of these changes was below the critical threshold established according to international food standards, but it could still impact the long‐term stability of the product.

Therefore, for the successful commercial implementation of the supplement powder produced under plasma treatment, long‐term storage and sensory evaluation on a larger scale are essential. Further research is recommended on systematic optimization, industrial‐scale validation, clinical trials, consumer acceptance, development of commercial applications, and regulatory frameworks.

The successful optimization of this process demonstrates the potential of CP to extend the shelf life of functional supplements made from marine sources while maintaining their nutritional and therapeutic benefits. This approach offers a promising alternative to traditional thermal methods and chemical treatments in industries that produce temperature‐sensitive functional foods.

## Author Contributions

**Hadis Amiri:** conceptualization, investigation, funding acquisition, writing – original draft, methodology, validation, writing – review and editing, visualization, software, formal analysis, project administration, data curation, supervision, resources. **Bahare Shabanpour:** funding acquisition, resources, investigation, visualization, conceptualization, project administration, supervision, writing – review and editing. **Parastoo Pourashouri:** conceptualization, supervision, writing – review and editing, investigation. **Samira Tajiknezhad:** conceptualization, writing – original draft, writing – review and editing, investigation.

## Ethics Statement

The sensory evaluation was conducted with approval from the Ethics Committee of the University. All sensory evaluators provided informed consent and followed the established guidelines.

## Conflicts of Interest

The authors declare no conflicts of interest.

## Data Availability

The data that support the findings of this study are available on request from the corresponding author. The data are not publicly available due to privacy or ethical restrictions.
